# Serum CD121a (Interleukin 1 Receptor, Type I): A Potential Novel Inflammatory Marker for Coronary Heart Disease

**DOI:** 10.1371/journal.pone.0131086

**Published:** 2015-06-22

**Authors:** Zhengxia Liu, Mengyao Zhang, Jin Wu, Ping Zhou, Ying Liu, Yucheng Wu, Yujiao Yang, Xiang Lu

**Affiliations:** Department of Geriatrics, the Second Affiliated Hospital, Nanjing Medical University, Jiangsu 210029, China; Charite Universitätsmedizin Berlin, GERMANY

## Abstract

Inflammation is now believed to be responsible for coronary heart disease (CHD). This belief has stimulated the evaluation of various inflammatory markers for predicting CHD. This study was designed to investigate the association between four inflammatory cytokines (CD121a, interleukin [IL]-1β, IL-8, and IL-11) and CHD. Here, we evaluated 443 patients with CHD and 160 CHD-free controls who underwent coronary angiography. Cytokines were evaluated using flow cytometry, and statistical analyses were performed to investigate the association between cytokine levels and the risk of CHD. Patients with CHD had significantly higher levels of CD121a. The odds ratios for CHD according to increasing CD121a quartiles were 1.00, 1.47 [95% confidence interval (CI): 0.79–2.72], 2.67 (95% CI: 1.47–4.84), and 4.71 (95% CI: 2.65–8.37) in an age- and sex-adjusted model, compared to 1.00, 1.48 (95% CI: 0.70–3.14), 2.25 (95% CI: 1.10–4.62), and 4.39 (95% CI: 2.19–8.79) in a model that was adjusted for multiple covariates. A comparison of the stable angina, unstable angina, and acute myocardial infarction (AMI) subgroups revealed that patients with AMI had the highest CD121a levels, although IL-1β levels were similar across all groups. IL-8 levels were also increased in AMI patients, and IL-11 levels were higher in CHD patients than in non-CHD patients. Correlation analysis revealed a positive association between CD121a, IL-8, and the Gensini score. Together, the significant increase in CD121a levels among CHD patients suggests that it may be a novel inflammatory marker for predicting CHD.

## Introduction

Cardiovascular disease (CVD) is the leading global cause of death and disability, and approximately half of these cases are directly attributed to coronary heart disease (CHD) [1]. Effective predictive and diagnostic methods are important for reducing the global burden of CHD on public health and its associated costs [2]. Currently, coronary angiography (CAG), coronary artery computed tomography, and intravascular ultrasound imaging are efficient but expensive approaches for diagnosing CHD. Therefore, a convenient and cheap method such as inflammatory biomarkers for predicting CHD is urgently needed.

As an important cause of CHD, inflammation is now widely believed to be a major mediator across the different stages of atherosclerosis, from initiation and advancement to plaque rupture and thrombosis [3,4]. Various inflammatory and biochemical markers have been implicated in atherogenesis, such as C-reactive protein (CRP), tumor necrosis factor-α, interleukin (IL)-6, IL-7, and IL-1β [5]. However, the roles of most inflammatory biomarkers remain unclear, and the role of these cytokines in the prediction of CHD has not been established.

Clinical and *in vivo* evidence accumulated over the previous years has suggested a pro-inflammatory role for IL-1β in atherosclerosis[6]. The type I IL-1 receptor (IL-1R1, CD121a) is the signaling binding receptors for IL-1β [7]. When IL-1β binds to CD121a, a signaling cascade is initiated that eventually leads to atherosclerosis [8,9]. In animal models, Chamberlain et al. have reported that Apoe(-/-)/CD121a(-/-) mice have significantly less atheroma than Apoe(-/-) mice [10]. In addition, Alexander et al. have reported that advanced atherosclerotic plaques in Apoe(-/-)/CD121a(-/-) mice unexpectedly exhibited plaque instability [11]. Unfortunately, despite these *in vivo* studies, only a few human studies have been conducted. In 2009, one clinical study reported that the levels of IL-1β and CD121a mRNA were significantly increased in atherosclerotic arteries (vs. normal arteries), although the authors failed to study the cytokines at the protein level [12]. In addition, the number of samples for the mRNA measurements in that study was rather small and these samples were extracted from the renal and carotid arteries, rather than the coronary arteries. Therefore, questions remain regarding the association between serum levels of CD121a and CHD.

IL-8 is a glycoprotein that belongs to the CXC subfamily and is considered to be primarily responsible for the recruitment of monocytes and neutrophils during inflammation [13]. The association between serum levels of IL-8 and CHD has been investigated in a few small studies with contradictory results [5,14]. IL-11 is a pleiotropic cytokine of the IL-6 family and is known to have multiple biological functions, including anti-inflammatory activity [15]. Although most studies have reported a protective role for IL-11 after acute myocardial infarction (AMI) [16], it is not known whether IL-11 is involved in plaque formation, development, and rupture in CHD.

Based on this information, we conducted a case-control study of 603 individuals to explore the association between CHD and levels of CD121a, IL-1β, IL-8, and IL-11.

## Methods

### Patients and controls

This study evaluated all individuals (29–88 years old, n = 987) who underwent CAG at the Cardiology Department of the Second Affiliated Hospital of Nanjing Medical University between March 2012 and April 2014. The exclusion criteria included coronary artery spasm angina, active inflammatory or infectious disease, malignant disease, autoimmune disease, valvular heart disease, severe hepatic and renal dysfunction, and recent surgery or injury. Patients with coronary stenosis less than 50% were also excluded. The included subjects (n = 603) were then divided according to their CAG results into two groups: a CHD group (443 patients with at least one coronary stenosis of >50% of the luminal diameter) and a control group (160 subjects who were totally free of coronary atherosclerosis). The CHD patients were further divided into 3 subgroups according to their clinical classification: stable angina (SA), unstable angina (UA), and AMI (Text A in S1 File). Gensini scores were calculated to evaluate the degree of coronary artery occlusion (Text B in S1 File).

The study design was approved by the Institutional Review Board of Nanjing Medical University, and written informed consent was obtained from all patients.

### Blood sampling and cytokine measurements

Blood samples were drawn into an inertia separation gel coagulation tube immediately after vascular puncture before CAG. CD121a, IL-1β, IL-8, and IL-11 levels were detected using the CBA Human Soluble Protein Detection Kit (BD Biosciences, US), as described in Text C in S1 File.

### Statistical analysis

Statistical analysis was performed using SPSS statistical software (version 19.0, SPSS, Chicago, IL). Normally distributed variables were expressed as mean ± standard deviation (SD), non-normally distributed variables were expressed as median (25th, 75th percentiles), and categorical variables were expressed as number (%). All cytokine values were non-normally distributed. The Mann-Whitney U test was used to compare the cases and controls, and the Kruskal-Wallis test was used to compare the CHD subgroups. Comparisons for all proportions were performed using Pearson’s χ^2^ test. We used unconditional logistic regression analyses to assess the association between CHD and the second, third, and fourth quartiles of each cytokine (compared to the first quartile), while simultaneously controlling for age and sex or multiple covariates consisting of age, sex, body mass index (BMI), smoking, alcohol consumption, hypertension, diabetes mellitus, hyperlipidemia, and statins use. Spearman correlation coefficients were calculated for the associations between the cytokine levels and the Gensini score or the various laboratory markers. The receiver operating characteristic (ROC) curves for the cytokines were used to evaluate their diagnostic accuracy for CHD. All tests were two-sided, and significance was set at *P* < 0.05.

## Results

### Clinical characteristics

Compared to controls, CHD patients were older; more likely to be male; and more likely to have hypertension, diabetes mellitus, and smoking or drinking habits. Among the laboratory markers, CHD patients also had higher levels of triglycerides, creatine kinase MB, cardiac troponin I, and myoglobin, and lower levels of high-density lipoprotein (HDL). A detailed comparison of the various groups is shown in Table 1.

**Table 1:** Clinical characteristics of the study population.

Characteristic	Controls (N = 160)	CHD cases (N = 443)	SA cases (n = 122)	UA cases (n = 271)	AMI cases (n = 50)
Demographics					
Age (years)	57.29 ±10.73	64.48±10.58*	64.25±12.00*	64.03±9.88*	67.50±10.32*
Males, n (%)	73 (45.6)	255 (57.6)*	69 (56.6)	154 (56.8)*	32 (64.0)*
BMI (kg/m^2^)	24.36 (22.51, 26.04)	24.66 (22.53, 26.04)	24.66 (22.89, 26.03)	24.66 (22.53, 26.12)	23.96 (21.12, 25.39)
Hypertension, n (%)	85 (53.1)	326 (73.6) *	88 (72.1) *	202 (74.5) *	36 (72.0) *
Diabetes mellitus, n (%)	21 (13.1)	129 (29.1) *	32 (26.2) *	70 (25.8) *	27 (54.0) * ^,^ ^#^ ^,^ ^§^
Smoking, n (%)	42 (26.3)	176 (39.7) *	44 (36.1)	111 (41) *	21 (42)
Alcohol consumption, n (%)	23 (14.4)	78 (17.6)	20 (16.4)	52 (19.2)	6 (12.0)
Statins use, n (%)	52 (32.5)	360 (81.3) *	70 (57.4) *	241 (88.9) * ^,^ ^#^	49 (98.0) * ^,^ ^#^
Laboratory markers					
TC (mmol/L)	4.37 (3.73, 5.04)	4.35 (3.64, 5.13)	4.15 (3.52, 4.87)	4.42 (3.68, 5.28)	4.44 (3.78, 5.34)
TG (mmol/L)	1.14 (0.84, 1.71)	1.37 (1.00,2.00) *	1.32 (0.91, 1.87)	1.44 (1.09, 2.03) *	1.31 (0.86, 1.80)
HDL (mmol/L)	1.15 (1.01, 1.35)	1.08 (0.91, 1.33) *	1.06 (0.92, 1.32) *	1.09 (0.9, 1.34) *	1.07 (0.89, 1.31)
LDL (mmol/L)	2.47 (1.96, 3.01)	2.48 (1.88, 3.08)	2.31 (1.87, 2.84)	2.53 (1.86, 3.12)	2.75 (2.10, 3.37) * ^,^ ^#^
CK-MB (ng/mL)	1.26 (0.7, 1.89)	1.59 (0.9, 2.6) *	1.22 (0.87, 2.35)	1.49 (0.81, 2.14)	4.10 (2.51, 15.68) * ^,^ ^#^ ^,^ ^§^
cTnI (×10^-3^, ng/mL)	5.00 (1.50, 10.00)	8.00 (1.50, 40.00) *	7.00 (1.50, 30.00)	6.00 (1.50, 20.00)	830.00 (120.00, 3450.00) * ^,^ ^#^ ^,^ ^§^
Mb (ng/mL)	30.94 (22.63, 48.12)	40.51 (28.56, 60.14) *	41.29 (29.11, 58.09) *	36.17 (27.18, 50.32) *	95.75 (41.76, 195) * ^,^ ^#^ ^,^ ^§^
hsCRP (mg/L)	3.00 (1.00, 6.00)	4.00 (1.00, 8.00)	3.00 (1.00, 6.25)	4.00 (1.00, 8.00)	7.00 (1.00, 15.00) *

CHD, coronary heart disease; SA, stable angina; UA, unstable angina; AMI, acute myocardial infarction; BMI, body mass index; TC, total cholesterol; TG, triglycerides; HDL, high-density lipoprotein; LDL, low-density lipoprotein; CK-MB, creatine kinase-MB; cTnI, cardiac troponin I; Mb, myoglobin; hsCRP, high-sensitivity C-reactive protein. Data are presented as mean ± standard deviation or median (25^th^ percentile, 75^th^ percentile).

*P < 0.05 vs. the control group.

^#^ *P* < 0.05 vs. the SA group.

^§^ *P* < 0.05 vs. the UA group.

**Fig 1:**
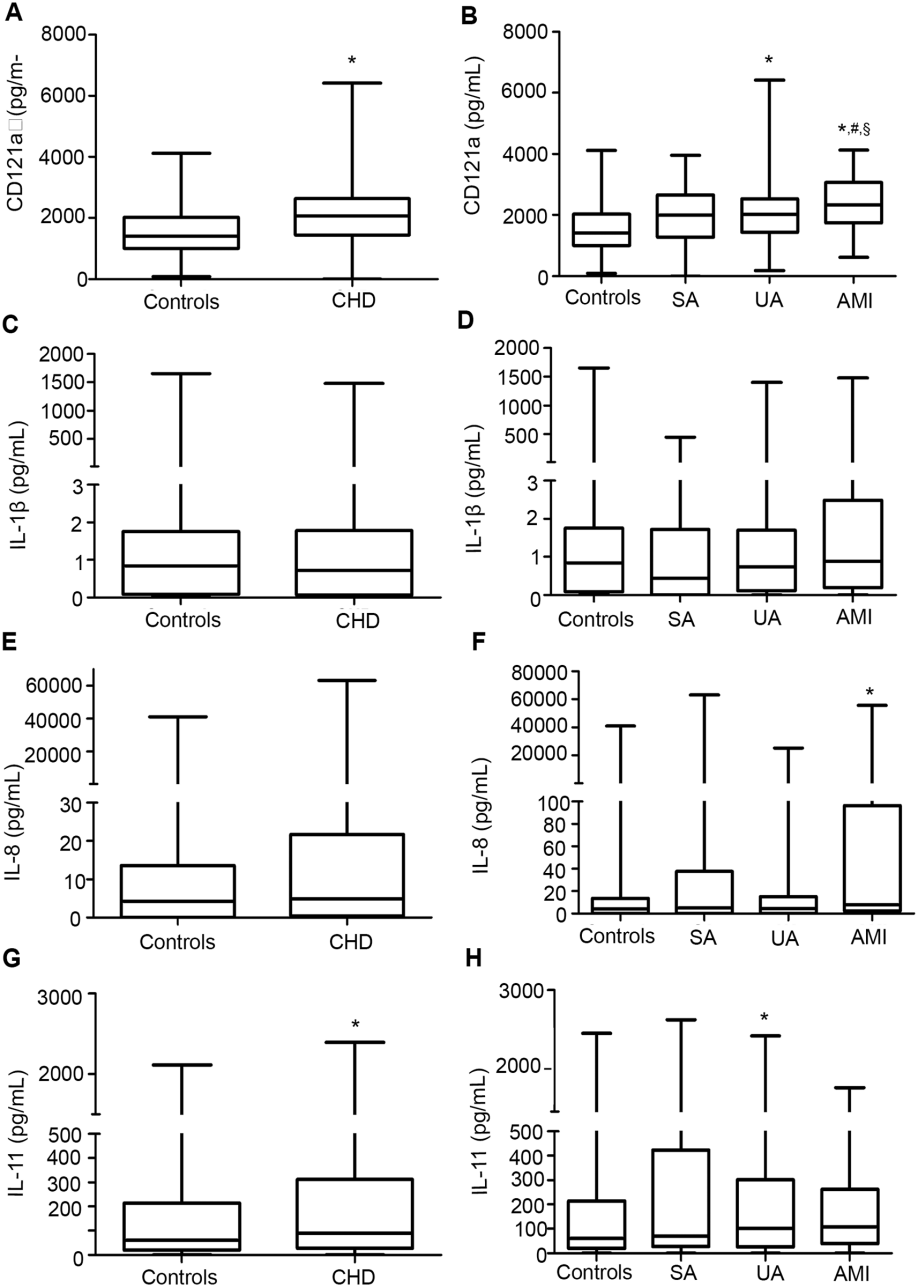
Comparison of cytokine levels among the various groups. (A) CD121a levels among patients with coronary heart disease (CHD) and controls. (B) CD121a levels among the CHD subgroups and controls. (C) IL-1β levels among patients with CHD and controls. (D) IL-1β levels among the CHD subgroups and controls. (E) IL-8 levels among patients with CHD and controls. (F) IL-8 levels among the CHD subgroups and controls. (G) IL-11 levels among patients with CHD and controls. (H) IL-11 levels among the CHD subgroups and controls. Values are medians (25^th^ and 75^th^ percentiles). *P < 0.05 vs. the control group, ^#^ *P* < 0.05 vs. the stable angina (SA) group, ^§^ *P* < 0.05 vs. the unstable angina (UA) group. AMI, acute myocardial infarction.

Interestingly, significantly higher levels of CD121a were observed in the CHD patients compared to the controls (Fig 1A). In addition, the SA, UA, and AMI subgroups all had significantly higher levels of CD121a compared to the controls (Fig 1B). Among the subgroups, patients with AMI had the highest CD121a levels. However, no differences in IL-1β levels were observed across the various groups (Fig 1C and 1D). Although patients with AMI had significantly increased levels of IL-8, the levels in the CHD group were similar to those in the control group (Fig 1E and 1F). Furthermore, significantly increased levels of IL-11 were observed in the CHD and UA groups compared to controls (Fig 1G and 1H).

### CHD risk in different quartiles of cytokine levels

The independent associations between serum cytokine levels and CHD are shown in Table 2. The second, third, and fourth quartiles of CD121a were associated with an increased risk of CHD, compared to the first quartile, although adjustment for age and sex or multiple covariates had minimal effect on the strength of this association. The OR for the highest CD121a quartile was 4.71 [95% confidence interval (CI): 2.65–8.37] in the age- and sex-adjusted model, compared to 4.39 (95% CI: 2.19–8.79) in the fully adjusted model. A small increase in the risk of CHD was observed for the highest quartile of IL-11 in the age- and sex-adjusted model, although this risk decreased in the fully adjusted model. No significant increase in the risk of CHD was observed for all levels of IL-1β and IL-8 in both models.

**Table 2:** Association between cytokine levels and the risk of CHD.

		OR and 95% CI			
Variable	Quartiles*	Adjusted for Age and Sex	*P* value	Adjusted for Multiple Covariates§	*P* value
CD121a	1st	1 reference		1 reference	
	2nd	1.47 (0.79–2.72)		1.48 (0.70–3.14)	
	3rd	2.67 (1.47–4.84)		2.25 (1.10–4.62)	
	4th	4.71 (2.65–8.37)	2.02×10^-7^	4.39 (2.19–8.79)	1.45×10^-4^
IL-1β	1st	1 reference		1 reference	
	2nd	1.02 (0.60–1.74)		0.67 (0.35–1.29)	
	3rd	0.76 (0.44–1.31)		0.67 (0.34–1.32)	
	4th	0.90 (0.53–1.55)	0.70	0.56 (0.29–1.08)	0.37
IL-8	1st	1 reference		1 reference	
	2nd	1.50 (0.85–2.64)		0.94 (0.47–1.86)	
	3rd	1.16 (0.68–1.96)		0.93 (0.49–1.77)	
	4th	1.24 (0.74–2.09)	0.56	0.82 (0.43–1.56)	0.94
IL-11	1st	1 reference		1 reference	
	2nd	1.12 (0.64–1.96)		1.18 (0.60–2.33)	
	3rd	1.43 (0.82–2.52)		1.68 (0.85–3.31)	
	4th	1.86 (1.09–3.17)	0.12	1.68 (0.89–3.19)	0.31

OR, odd ratio; CI, confidence interval.

*The quartiles were defined based on the distribution of cytokines in the control patients, and the lowest quartile was used as the reference value.

^§^Adjusted for age, sex, body mass index, smoking, alcohol consumption, hypertension, diabetes mellitus, hyperlipidemia, and statins use.

### Cytokine levels and related risk factors

The levels of the four cytokines in control subjects were further evaluated according to various sociodemographic categories in S2 File. Significant differences in cytokine levels were observed according to age (CD121a), BMI (IL-1β), hypertension (IL-1β and IL-8), smoking (CD121a), and alcohol consumption (CD121a and IL-1β).

### Association of cytokine levels with Gensini score and laboratory markers

**Fig 2:**
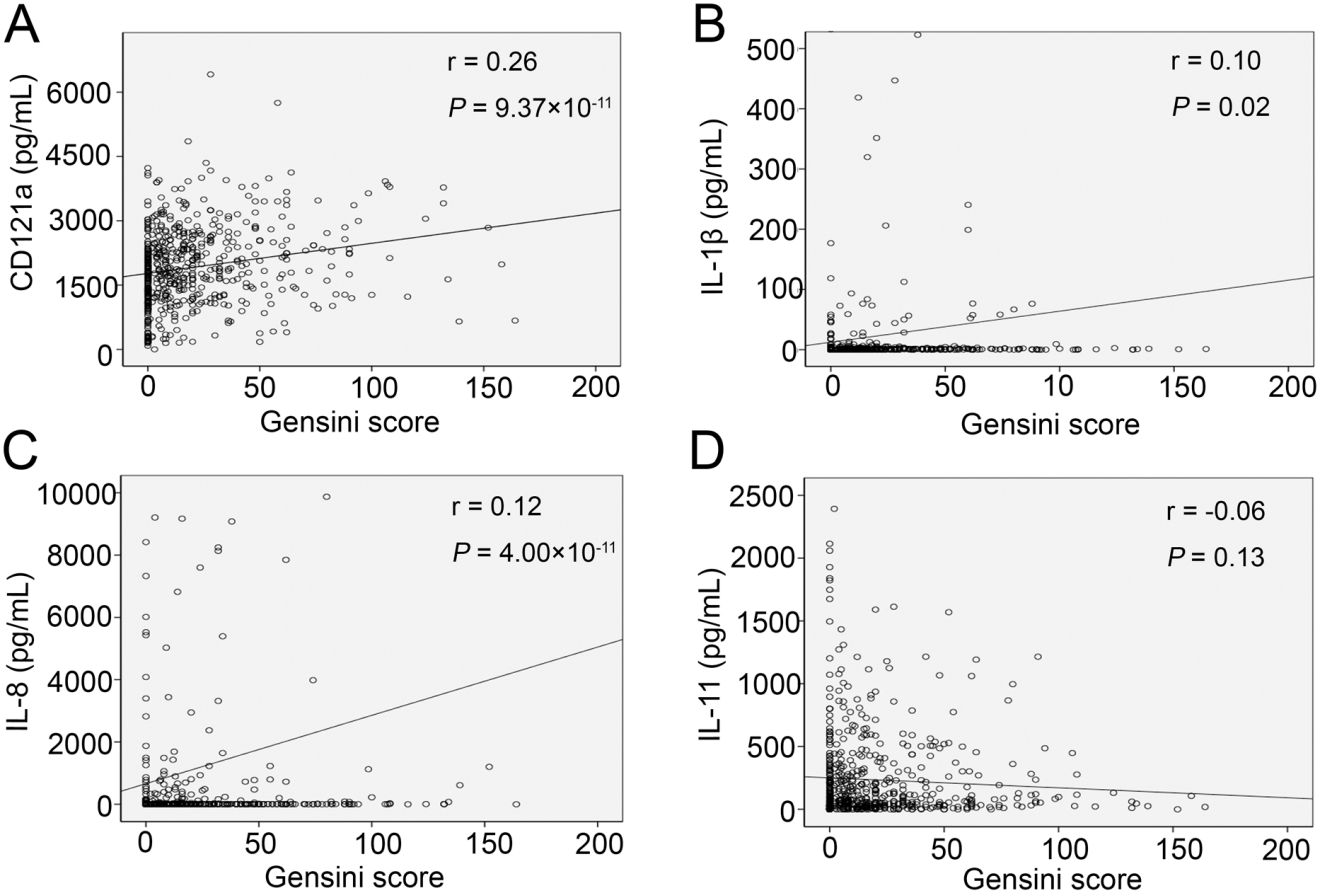
Spearman rank correlations between the various cytokines and the Gensini score. (A) CD121a, (B) IL-1β, (C) IL-8, and (D) IL-11.

We analyzed the association between the cytokines and Gensini score or established laboratory markers for CHD. Positive correlations were observed between the Gensini score and CD121a (r = 0.26, *P* = 9.37×10^-11^) and IL-8 (r = 0.12, *P* = 4.00×10^-3^), although no associations were observed for IL-1β and IL-11 (Fig 2). CD121a was positively associated with creatine kinase MB, cardiac troponin I, and myoglobin, and was negatively associated with total cholesterol and HDL (*P* < 0.05), shown in S3 File.

### Diagnostic accuracy for CHD of the various cytokines

**Fig 3:**
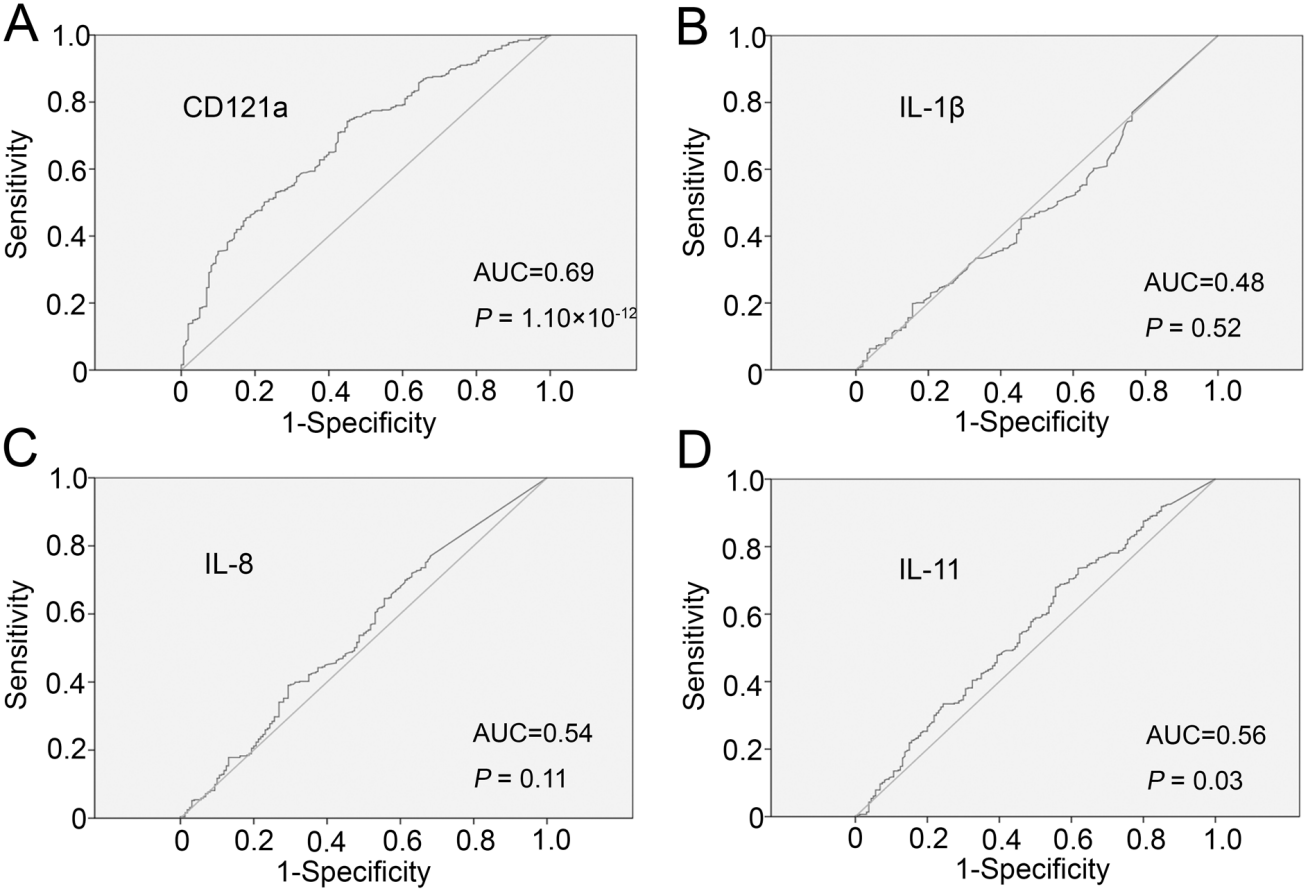
Receiver operating characteristic curves for the diagnostic accuracy of cytokines for coronary heart disease. (A) CD121a, (B) IL-1β, (C) IL-8, and (D) IL-11. AUC, area under the curve.

Finally, we conducted ROC curve analysis to identify the diagnostic accuracy of these cytokines for CHD. A CD121a level of 1,487.84 pg/mL demonstrated 74.3% sensitivity and 55% specificity (Fig 3A) for CHD, and an IL-11 level of 42.42 pg/mL demonstrated 67.9% sensitivity and 44.4% specificity (Fig 3D) for CHD. The areas under the curve for CD121a and IL-11 in CHD were 0.69 and 0.56, respectively. IL-1β and IL-8 had no significantly diagnostic value for CHD (Fig 3B and 3C).

## Discussion

In this case-control study, we observed a significant positive association between serum levels of CD121a and the risk of CHD, which persisted after adjusting for various CHD risk factors in different models. A trend of increasing CD121a levels with increasing CHD severity was observed, and this trend reached significance in the AMI subgroup. These results indicated that CD121a may be taken as a risk factor for CHD.

IL-1 is a classic pro-inflammatory cytokine that is linked to atherogenesis and has been studied extensively [17]. Unlike IL-1α, which binds to the membrane of the producing cell and exerts a local effect, IL-1β is secreted and circulates systemically [8]. Therefore, IL-1β is easier to detect and is thought to be more relevant to atherosclerosis-related inflammation [18]. The binding receptors for IL-1β include CD121a, which is responsible for triggering IL-1-mediated inflammation, and the type II IL-1 receptor, which acts as non-signaling decoy receptor [7]. Our results are consistent with those of previous studies that have reported elevated CD121a mRNA in atherosclerotic arteries [12] and CD121a(-/-) mice with reduced atheroma [10]. As CD121a is the only signaling membrane receptor for IL-1β, elevated CD121a may be responsible for activating IL-1-mediated inflammation in CHD [7,19]. It is reported that the loss of IL-1 signaling in CD121a(-/-) mice results in globally attenuated leukocyte recruitment and reduced infiltration of pro-inflammatory leukocytes into the infarcted myocardium [20]. However, we unexpectedly failed to find a significant association between IL-1β and CHD (or with the various subgroups), which appears to conflict with the documented role of IL-1β in nearly all phases of atherosclerosis. For example, Waehre et al. have observed increased levels of IL-1β mRNA in peripheral blood mononuclear cells from patients with SA and UA [21]. In addition, IL-1β can augment megakaryocyte and platelet function to promote atherothrombosis via CD121a [22], and Bhaskar et al. have reported that an anti-IL-1β antibody can inhibit the progression of atherosclerosis *in vivo* [23]. The Canakinumab Anti-inflammatory Thrombosis Outcomes Study (CANTOS) is also attempting to use canakinumab (a monoclonal anti-IL-1β antibody) to evaluate the inflammatory hypothesis of cardiovascular disease, as well as the critical role of IL-1β-mediated inflammation in the development of cardiovascular diseases, including CHD [24]. In our study, the levels of IL-1β in the CHD groups were similar to those of controls, similar to the findings of Olofsson et al., who reported no significant difference in the protein levels of IL-1β in atherosclerotic and normal arteries, despite the obvious association between IL-1β mRNA and atherosclerosis [12]. Later studies have described three forms of IL-1β, which include pro-IL-1β (31 kDa), intermediate IL-1β (28 kDa), and mature IL-1β (17 kDa) [25]. Cholesterol crystals can promote maturation of pro-IL-1β into IL-1β via biologically activation [26,27]. Intriguingly, a recent study has reported that statins can process pro-IL-1β into the intermediate form, which cannot activate CD121a and interferes with the mature IL-1β-CD121a signaling [25]. In our study, a large number of subjects were taking statins; therefore we speculate that our serum protein levels of IL-1β actually contained the three forms of IL-1β. Thus, developing a clinical method for detecting only mature IL-1β may further improve the predictive value of this cytokine for CHD. In addition, blockade of angiotensin II type 1 receptors has been reported to reduce excessive IL-1β production and release [28]. As the majority of CHD patients in our study had hypertension and were taking angiotensin receptor blockers, these factors may also have partially influenced the IL-1β levels measured in this study. However, despite these potential factors, CD121a remained significant increase in CHD patients than controls, while IL-1β levels were similar between the two groups. Thus, CD121a tended to be a better and more stable serum marker for predicting CHD compared to IL-1β.

We observed increased levels of IL-8 in AMI patients compared to those in controls, as well as a positive association between IL-8 and the Gensini score; these results confirmed previous reports in which IL-8 concentrations were increased in AMI patients [29]. Since its discovery during the late 1980s, IL-8 has been described as a participant in all stages of atherosclerosis [30] and facilitator of plaque vulnerability and disruption [5,31]. However, we only observed increased levels of IL-11 in the CHD and UA groups, compared to those in the controls. We speculate that increased IL-11 might play a cardioprotective role as a reactive response to atherosclerosis in CHD. Similarly, Obana et al. observed that expression of IL-11 mRNA was upregulated in the heart after myocardial infarction, and demonstrated that IL-11 attenuated adverse cardiac remodeling in those cases [32].

To the best of our knowledge, this study is the first to demonstrate that serum levels of CD121a are elevated in CHD patients, particularly among AMI patients, and that increasing CD121a levels are associated with an increasing risk of CHD. What’s more, it appears that CD121a is less affected by various therapies and is more stable at the serum level. Therefore, we believe that CD121a may help to predict CHD. Several limitations existed in our study. Firstly, parameters such as myocardial enzymes were measured in different time, contributing to our poor or even negative correlations between these parameters and CD121a levels. What’s more, whether the expressions of CD121a also have dynamic changes needs further investigation. If so, results of ROC curve analysis of this cytokine may be improved by following up its dynamic expression. Secondly, CD121a, as an inflammatory receptor for IL-1, also plays a pro-inflammatory role in other disease such as pulmonary inflammation [33,34], which could be responsible for the low specificity for diagnosing CHD. In addition, the sample size especially for AMI group may also be a shortage of our study.

## Conclusions

In conclusion, our results highlighted the increased level of serum CD121a in CHD patients than controls and established a potential correlation between levels of CD121a and the severity of CHD. These findings suggest that CD121a may be a novel inflammatory biomarker for prediction of CHD. To further elucidate the value of CD121a in CHD, repeated CD121a measurements in a larger-scale study and mechanistic experiments on association in atherosclerosis and this cytokine are warranted.

## Supporting Information

S1 File Supplementary methods. (DOC)

S2 File Mean serum cytokine levels according to various sociodemographic variables in the control subjects. (DOC)

S3 File Spearman rank correlations between cytokines and the established laboratory markers for coronary heart disease. (DOC)
