# Supplementary material for: Serum CD121a (Interleukin 1 Receptor, Type I): A Potential Novel Inflammatory Marker for Coronary Heart Disease
Source: PLoS One. 2015 Jun 22;10(6):e0131086. doi: 10.1371/journal.pone.0131086 (PMC4476662; doi:10.1371/journal.pone.0131086)
Supplement: S1 File — (DOC) [file pone.0131086.s001.doc]

**Text A. Clinical classification of coronary heart disease.**

The stable angina (SA) subgroup included patients with typical effort angina that was accompanied by a downward or horizontal ST-segment depression of >1 mm during an exercise test, as well as angiographically documented obstructive coronary artery disease (CAD). The unstable angina (UA) subgroup included patients who exhibited chest pain at rest, with definite ST-segment changes and/or T-wave inversions and angiographically documented obstructive CAD. The acute myocardial infarction (AMI) subgroup contained patients who exhibited significantly elevated levels of creatine kinase MB (CK-MB) and cardiac troponin I (cTnI), as well as a typical clinical electrocardiogram manifestation.

**Text B. Criterion of Gensini scores.**

Scores of 1, 2, 4, 8, 16, and 32 were assigned to stenoses (expressed as a percentage of the luminal diameter) of 25%, 50%, 75%, 90%, 99%, and 100%, respectively. The score was then was multiplied by 5 (left main branch), 2.5 (proximal left anterior descending branch or proximal left circumflex branch), 1.5 (mid-left anterior descending branch), 0.5 (second diagonal branch or left posterolateral branch), or 1 (all other branches). After the score for each lesion was calculated, the scores were added to calculate the severity score for the patient’s entire coronary system.

**Text C. Cytokine measurements.**

Cytokines were measured using the CBA Human Soluble Protein Detection Kit (BD Biosciences, US). Samples and standards (50 μL each) were incubated in centrifuge tubes with capture beads for 1 h at room temperature in the dark. The phycoerythrin detection reagent was then added to each tube for an additional 2 h of incubation at room temperature in the dark. The samples were washed and the bead pellets were re-suspended in washing buffer. The re-suspended samples were then run on a flow cytometer (FACS Canto II, BD Biosciences, US) that was equipped with BD Diva software. Two thousand events in the gated bead population were collected, and 5-parameter data were saved for subsequent analysis using BD FCAP Array software. Serum concentrations were derived using the standard curve and expressed in pg/mL.
