# Supplementary material for: Serum CD121a (Interleukin 1 Receptor, Type I): A Potential Novel Inflammatory Marker for Coronary Heart Disease
Source: PLoS One. 2015 Jun 22;10(6):e0131086. doi: 10.1371/journal.pone.0131086 (PMC4476662; doi:10.1371/journal.pone.0131086)
Supplement: S2 File — (DOC) [file pone.0131086.s002.doc]

**S2 Table.** Mean serum cytokine levels according to various sociodemographic variables in the control subjects.

|  |  | CD121a | IL-1β | IL-8 | IL-11 |
| --- | --- | --- | --- | --- | --- |
| Ages, years | < 65 (n=122) | 1377.17 (959.98, 1918.49) * | 0.84 (0.10, 1.77) | 4.14 (0, 9.71) | 57.69 (20.87, 234.23) |
|  | ≥ 65 (n =38) | 1517.86 (1097.12, 2311.33) * | 0.82 (0, 1.67) | 6.01 (0, 124.62) | 74.78 (18.69, 214.02) |
| Sex | Male (n =73) | 1354.36 (889.20, 1833.17) | 0.84 (0, 1.59) | 3.96 (0, 29.53) | 51.96 (13.16, 198.56) |
|  | Female (n =87) | 1698.20 (1036.88, 2078.30) | 0.84 (0.14, 1.79) | 4.31 (0, 8.93) | 75.16 (22.12, 275.36) |
| BMI | < 25 (n =105) | 1405.50 (1009.06, 2029.58) | 0.69 (0, 1.60) * | 4.59 (0, 11.49) | 63.70 (20.01, 255.03) |
|  | ≥ 25 (n =55) | 1419.68 (962.52, 1911.74) | 1.28 (0.31, 2.28) * | 2.82 (0, 27.76) | 59.08 (20.02, 214.02) |
| Hypertension | Absent (n =75) | 1560.52 (1025.88, 2054.68) | 0.64 (0, 1.4) * | 1.59 (0, 6.83) * | 75.16 (18.16, 279.48) |
|  | Present (n =85) | 1358.48 (962.52, 1904.63) | 1.28 (0.32, 2.15) * | 5.93 (0, 92.77) * | 57.78 (21.98, 209.47) |
| Diabetes mellitus | Absent (n=139) | 1377.17 (1015.04, 1938.72) | 0.85 (0.10, 1.81) | 4.31 (0, 12.51) | 59.08 (20.02, 214.02) |
|  | Present(n =21) | 1809.72 (756.16, 2265.05) | 0.64 (0, 1.63) | 2.25 (0, 19.7) | 114.15 (22.15, 356.28) |
| Smoking | Absent (n =118) | 1705.83 (1074.83, 2151.53) * | 0.85 (0.17, 1.75) | 4.45 (0, 10.24) | 68.53 (20.87, 222.62) |
|  | Present (n =42) | 1135.70 (452.68, 1380.05) * | 0.72 (0, 2.13) | 2.82 (0, 85.73) | 54.20 (16.32, 206.39) |
| Alcohol consumption | Absent (n =137) | 1531.30 (1050.35, 2090.25) * | 0.81 (0, 1.60) * | 3.96 (0, 9.75) | 63.70 (19.44, 214.02) |
|  | Present (n =23) | 1088.83 (295.68, 1388.68) * | 1.40 (0.40, 8.68) * | 7.23 (0, 1413.06) | 40.20 (21.84, 325.08) |
| Hyperlipidemia | Absent (n =80) | 1426.89 (1018.14, 1961.36) | 0.85 (0.05, 1.69) | 4.00 (0, 14.80) | 92.63 (23.32, 242.79) |
|  | Present (n =58) | 1601.53 (1043.61, 2105.58) | 0.88 (0.24, 2.70) | 5.55 (0, 52.54) | 49.93 (16.75, 218.14) |

BMI, body mass index. Data are presented as median (25^th^ percentile, 75^th^ percentile).

**P* < 0.05 for intra-category comparisons.
