# Supplementary material for: Serum CD121a (Interleukin 1 Receptor, Type I): A Potential Novel Inflammatory Marker for Coronary Heart Disease
Source: PLoS One. 2015 Jun 22;10(6):e0131086. doi: 10.1371/journal.pone.0131086 (PMC4476662; doi:10.1371/journal.pone.0131086)
Supplement: S3 File — (DOC) [file pone.0131086.s003.doc]

**S3 Table.** Spearman rank correlations between cytokines and the established laboratory markers for coronary heart disease.

|  | CD121a | IL-1β | IL-8 | IL-11 |
| --- | --- | --- | --- | --- |
| TC | -0.12 | 0.01 | 0.05 | 0.07 |
| *P* value | 0.01 | 0.76 | 0.25 | 0.15 |
| TG | -0.02 | -0.01 | 0.02 | -0.08 |
| *P* value | 0.74 | 0.83 | 0.69 | 0.09 |
| HDL | -0.17 | 0.08 | 0.08 | 0.11 |
| *P* value | 1.52×10^-4^ | 0.07 | 0.08 | 0.02 |
| LDL | -0.07 | 0.03 | 0.03 | 0.03 |
| *P* value | 0.12 | 0.58 | 0.46 | 0.53 |
| CK-MB | 0.33 | -0.14 | -0.07 | -0.11 |
| *P* value | 7.80×10^-13^ | 3.00×10^-3^ | 0.13 | 0.02 |
| cTnI | 0.18 | -0.09 | -0.05 | 0.04 |
| *P* value | 8.13×10^-5^ | 0.05 | 0.34 | 0.41 |
| Mb | 0.15 | 0.08 | 0.06 | -0.02 |
| *P* value | 2.00×10^-3^ | 0.11 | 0.18 | 0.72 |
| hsCRP | 0.06 | 0.02 | -0.05 | 0.06 |
| *P* value | 0.27 | 0.72 | 0.28 | 0.26 |

TC, total cholesterol; TG, triglycerides; HDL, high-density lipoprotein; LDL, low-density lipoprotein; CK-MB, creatine kinase-MB; cTnI, cardiac troponin I; Mb, myoglobin; hsCRP, high-sensitivity C-reactive protein.
